# A randomized phase 3 trial comparing paclitaxel plus 5-fluorouracil versus cisplatin plus 5-fluorouracil in Chemoradiotherapy for locally advanced esophageal carcinoma—the ESO-shanghai 1 trial protocol

**DOI:** 10.1186/s13014-018-0979-0

**Published:** 2018-02-27

**Authors:** Yun Chen, Zhengfei Zhu, Weixin Zhao, Ling Li, Jinjun Ye, Chaoyang Wu, Huarong Tang, Qin Lin, Jiancheng Li, Yi Xia, Yunhai Li, Jialiang Zhou, Kuaile Zhao

**Affiliations:** 1 Department of Radiation Oncology, Fudan University Shanghai Cancer Center, 270 DongAn Road, Shanghai, 200032 China; 2 Jiangsu Cancer Hospital, Nanjing, China; 3 Zhenjiang First People’s Hospital, Zhenjiang, China; 4 The First Affiliated Hospital of Xiamen University, Xiamen, China; 5 Fujian Provincial Cancer Hospital, Fuzhou, China; 6 Fudan University Shanghai Cancer Center Minhang Branch, Shanghai, China; 7 Affiliated Hospital of Jiangnan University, Wuxi, China

**Keywords:** Esophageal squamous cell carcinoma, Concurrent chemoradiotherapy, Paclitaxel, Cisplatin, 5-fluorouracil

## Abstract

**Background:**

Concurrent chemoradiotherapy is a standard modality for locally advanced esophageal squamous cell carcinoma (ESCC) patients. Cisplatin combined with 5-fluorouracil continuous infusion (PF) remains the standard concurrent chemotherapy regimen. However, radiotherapy concurrent with PF showed a high incidence of severe side effects. Paclitaxel showed a promising radiosensitivity enhancement in the treatment of esophageal carcinoma in both vitro and vivo studies. The ESO-Shanghai 1 trial examines the hypothesis that paclitaxel plus 5-fluorouracil (TF) concurrent with radiotherapy has better overall survival and lower toxicity for patients with local advanced ESCC.

**Method:**

Four hundred thirty-six ESCC patients presenting with stage IIa to IVa will be enrolled in a prospective multicenter randomized phase 3 study. Patients will be randomized to either concurrent chemoradiotherapy with PF (cisplatin 25 mg/m^2^/d, d1–3, plus 5-fluorouracil 1800 mg/m^2^, continuous infusion for 72 h) once every 4 weeks for 2 cycles followed by consolidation chemotherapy for 2 cycles or concurrent chemoradiotherapy with weekly TF (5-fluorouracil 300 mg/m^2^, continuous infusion for 96 h plus paclitaxel 50 mg/m^2^, d1) for 5 weeks followed by consolidation chemotherapy (5-fluorouracil 1800 mg/m^2^, continuous infusion for 72 h, plus paclitaxel 175 mg/m^2^ d1) once every 4 weeks for 2 cycles. The radiotherapy dose is 61.2 Gy delivered in 34 fractions to the primary tumor including lymph nodes. The primary end-point is the 3-yr overall survival analyzed by intention to treat. The secondary endpoints are disease progression-free survival, local progression-free survival, and number and grade of participants with adverse events.

**Discussion:**

The aim of this phase 3 study is to determine whether the TF regimen could replace the standard PF regimen for inoperable ESCC patients. An overall survival benefit of 12% at 3 years should be expected in the TF group to achieve this goal.

**Trial registration:**

ClinicalTrials.gov Identifier: NCT01591135. Registered 18 April 2012.

## Background

Concurrent chemoradiotherapy (CCR) is a standard modality for locally advanced esophageal squamous cell carcinoma (ESCC) patients. Cisplatin combined with 5-fluorouracil continuous infusion (PF) remains the standard concurrent chemotherapy regimen [1]. Identifying a new combination of drugs with superior efficacy but less toxicity is one of the important solutions to enhance radiosensitivity to improve the survival of this disease. Recently, both experimental and clinical research has demonstrated good efficacy and radiosensitivity of paclitaxel (PTX) in the treatment of esophageal carcinoma (EC). As a result, many researchers and oncologists have shown a great interest in the role of this promising agent played in CCR in EC.

### PTX is one of the effective agents in the treatment of EC

Early in 1994, the co-work at the University of Texas M.D. Anderson Cancer Center (MDACC) and Memorial Sloan-Kettering Cancer Center proved that PTX is one of the effective agents in the treatment of EC, with the response rate of 34% in adenocarcinoma and 28% in squamous cell carcinoma [2, 3]. Thereafter, many institutions have conducted numerous studies using PTX combined with other agents, among which PTX plus cisplatin (TP) and PTX plus cisplatin plus 5-fluorouracil (TPF) were the most common combinations used in the treatment of advanced EC, with a response rate of 41% to 55% (Table 1)**.**

**Table 1:** Efficacy of PTX-based chemotherapy for advanced esophageal cancer

Authors	Cases (SC/AC/other)	Chemotherapy	Efficacy		
			Total	SC	AC
van der Gaast, et al. [20]	31(NA/NA)	PTX 100–160 mg/m^2^ iv3h, d1; DDP 60 mg/m^2^, d1, q2w	55%	53%	60%
Kelsen, et al. [21]	37(27/10)	PTX 200 mg/m^2^, civ24h, d1; DDP 75 mg/m^2^, d1, q3w	49%	60%	44%
Polee, et al. [22]	51(16/31/4)	PTX 180 mg/m^2^, iv3h, d1; DDP 60 mg/m^2^, d1, q2w	43%	39%	44%
Huang, et al. [23]	30(30/0)	PTX 175 mg/m^2^, iv3h, d1; DDP 40 mg/ m^2^, d2, 3, q3w	59%	59%	–
Ilson, et al. [24]	61(31/30)	PTX 175 mg/m^2^ iv3h, d1; DDP 20 mg/m^2^ 5d; 5-FU 1 g/m^2^/d, 5d; q3w	48%	50% (CR 20%)	46%(CR 3%)
Lin, et al. [25]	41(41/0)	PTX 35 mg/m^2^, d1, 4, 8, 11, DDP 20 mg/m^2^, d2, 5, 9, 12; 5-FU 2 g/m^2^, q3w	39%	39%	–

*Abbreviations*: *SC* squamous cell carcinoma, *AC* adenocarcinoma, *PTX* paclitaxel, *5-FU* 5-fluorouracil, *DDP* cisplatin, *CR* complete response

### Preclinical research shows the radiosensitivity enhancement of PTX

PTX has two main antitumor mechanisms: ① blocking the tumor at the G2/M phase of the cell cycle so as to inhibit the cell mitosis and proliferation; ② inducing the apoptosis of tumor cells. The inhibition of mitosis occurs quickly and disappears soon after, while apoptosis occurs late and last for a long time. Mitosis inhibition appears 2 h after the intravenous infusion of PTX and then accumulates over time. It reaches its peak after 8 to 12 h and is reduced gradually, and then it disappears in 1 to 2 days. By contrast, apoptosis occurs 6 to 9 h after the drug administration and gradually reaches a peak in 12 to 24 h that lasts for several hours to 2 days before gradually being reduced to the baseline after 2 to 3 days. Inhibition of mitosis does not cause the death of cells, but apoptosis does [4, 5]. Based on these mechanisms, researchers have tried to optimize the regimen of CCR using PTX to exaggerate the radiosensitivity enhancement.

Regarding in vitro experiments, most studies have found that PTX has the supra-additive action with the enhancement ratio of 1.1 to 3.4 [4]. However, some studies have shown only additive action or even subadditive action of PTX. An explanation for the different outcomes may be the different cell lines, proliferation status, drug concentration, or timing of administration of drug and irradiation chosen in different studies [6]. The studies showing a high ratio of radiosensitivity enhancement of PTX mostly used cell lines with high proliferation potential, drug concentrations of 5 to 100 nmol/L, cell lines that were cultured for more than 24 h, and radiation when most cells reached the G2/M phase of the cell cycle after the administration of PTX. As a result, these conditions would help radiation killed the cells so as to improve the effect.

Regarding in vivo experiments, PTX also showed a good radiosensitivity enhancement with a ratio of 1.2 to 2.0. However, the highest radiosensitivity ratio did not occur at the time when most cells were arrested at the G2/M phase after irradiation as in vitro studies, but it occurred 3 days after the administration of PTX to the irradiated tumor cells. The radiosensitivity ratio is then decreased 4 days after the administration of PTX. The results of the in vitro and in vivo studies were obviously different and may demonstrate unknown mechanisms other than mitosis inhibition of PTX. The percentage of hypoxia cells in tumor cells was 32% before drug administration and decreased to 15%, 4%, 2% and 1% after 9, 24, 48, and 72 h of PTX administration, respectively. The tendency exactly matched the change in the radiosensitivity ratio. Thus, the reoxygenation of hypoxia cells induced by apoptosis play a more important role than mitosis inhibition in the mechanism of radiosensitivity enhancement of PTX in treating solid tumors [7, 8].

Consequently, PTX played a radiosensitivity enhancement role in the laboratory studies. However, only by using certain cell lines, an adequate PTX concentration and the appropriate irradiation timing could we obtain the expected results. Otherwise, PTX may play no role in radiosensitivity enhancement and may even counteract the radiation effects and decrease the therapeutic effects. Unfortunately, to date, we have found no study that has focused on the radiosensitivity enhancement role of PTX played in EC cell lines.

### Phase 2 clinical trials of EC patients treated with PTX-based CCR

Neoadjuvant CCR followed by surgery is the standard treatment for operable EC patients, especially in western countries. Previous randomized studies have shown that the pathologic complete response (pCR) and 3-yr overall survival (OS) of PF concurrent with 40 Gy of radiotherapy was 15–40% and 26–32%, respectively [9]. Since PTX had considerable efficiency and radiosensitivity enhancement in the preclinical studies, researchers conducted many clinical studies with different PTX-based concurrent regimens to find an optimal one to replace the PF regimen. In 1997, Massachusetts General Hospital and Fox Chase Cancer Center separately reported a phase 1 study of neoadjuvant CCR with PTX-based regimen followed by surgery in EC patients [10, 11]. The results confirmed that the TPF regimen was tolerable and efficient with a pCR rate of up to 39% in operable EC patients and 50% in patients who could complete all the chemotherapy cycles. Over the past decade, researchers from different countries and institutions have performed many phase 2 studies based on this phase 1 study. Although there were some differences in the dose and combination of PTX among the different studies, the pCR rates of the PTX-based regimen were 16–67% higher than the classical PF regimen (Table 2).

**Table 2:** Phase 2 studies of PTX-based preoperative neoadjuvant chemoradiotherapy for EC patients

Authors	Cases (SC/AC/other)	Chemotherapy	Radiation dose (Gy)	Median survival (months)	pCR		
					Total	SC	AC
Safran, et al. [26]	41 (12/29)	PTX 60 mg/m^2^ 3 h; DDP 25 mg/m^2^ d1, 8, 15, 22	39.6	15	29%	42%	24%
Bains, et al. [27]	41 (16/25)	Induction, DDP 75 mg/m^2^; PTX 175 mg/m^2^, W 1, 4; Concurrent, DDP 30 mg/m^2^/w; PTX 30–80 mg/m^2^/w, 96 h civ w 7–12	50.4	–	22%	31%	16%
Urba, et al. [28]	69 (10/57/2)	DDP 75 mg/m^2^ d1; PTX 60 mg/m^2^ d1, 8, 15, 22	45	24	19%	–	–
Van Meerten, et al. [29]	54 (12/41/1)	PTX 50 mg/m^2^, d1, 8, 15, 22, 29; CBP AUC = 2, d1, 8, 15, 22, 29	41.4	–	25%	–	–
Meluch, et al. [30]	129 (35/91/3)	PTX 200 mg/m^2^, 1 h, d1, 22; CBP AUC = 4, d1, 22; 5-FU 225 mg/m^2^/d, civ, d1–42	45	22	38%	53%	37%
Lin, et al. [31]	97 (92/5)	PTX 35 mg/m^2^, 1 h, d1, d4; DDP 15 mg/m^2^ d2, d5	40	29	25%	–	–
Jatoi, et al. [32]	54 (−/−)	PTX 200 mg/m^2^, 1 h, d1, 22; CBP AUC = 4, d1, 22; 5-Fu 225 mg/m^2^/d, civ, d1–42	45	21.2	35%	–	–

*Abbreviations*: *SC* squamous cell carcinoma, *AC* adenocarcinoma, *pCR* pathologic complete response, *PTX* paclitaxel, *5-FU* 5-fluorouracil, *DDP* cisplatin, *CBP* carboplatin, *AUC* area under the curve

### Case-control study of PTX-based regimen versus PF regimen in CCR in EC patients

Based on the preferable results of phase 2 studies, PTX was widely used in clinical practice. However, there was no prospective randomized phase 3 study comparing the PTX-based regimen with the PF regimen in CCR in EC patients. Recently, several retrospective non-randomized case-control studies compared the toxicities between these two regimens. Unfortunately, the outcome was disappointing. In 2000, the Cleveland Clinic Foundation initiated a phase 2 study of neoadjuvant CCR with the PTX-based regimen followed by surgery in EC patients, and historically compared the study with another conducted at the same institution using the PF regimen instead [12]. The results showed that, although the pCR rate and 3-yr OS in both studies were similar, the incident rate of severe leukocytopenia and the hospital stay time in the PTX-based group were twice than that in the PF group. Because these two studies were performed at the same institute using similar inclusion criteria, and had the same radiotherapy prescriptions and comparable clinical characteristics such as stages and pathologies, the results were partially reliable despite the limitations of comparative historical research. Certainly, some researchers had different opinions and queried the design of the study with non-randomized, insufficient cases, no standard treatment, and unreasonable split course of preoperative chemoradiotherapy [13]. Therefore, this negative result did not stop the researchers from further investigating PTX in CCR for the treatment of EC. However, many retrospective studies showed no significant difference in both the efficiency and side effects between the two regimens (Table 3).

**Table 3:** Case-control studies of the comparison between the PTX-based regimen and the PF regimen in preoperative neoadjuvant chemoradiotherapy

Institution/ Study Type	Cases	CT	RT	pCR (%)			3-yr OS (%)	≥3 Side effect
				Total	SC	AC		
Cleveland Clinic Foundation [12]/ prospective, non-randomized	72	PF	45 Gy, 1.5 Gy/FX, BID (split course) or 24 Gy postoperative	27	36	22	36	Vomiting (1%), mucositis (18%), leucopenia (43%), thrombocytopenia (10%), nephrotoxicity (8%), unplanned hospitalization (25%)
	40	TP		23	50	8	30	Mucositis (13%), leucopenia (95%), neuropathy (13%), unplanned hospitalization (48%)
Duke University [9]/ retrospective	57	PF	45–50.4 Gy, 1.8–2.0 Gy/FX	40	–	–	37	24%
	52	TPF		39	–	–	37	34%
Massachusetts General Hospital [33]/ retrospective	81	PF	CTV 45 Gy/25FX, GTV 58.5 Gy/25FX (13.5 Gy, 1.5 Gy/FX concomitant boost during the first and second cycles of CT)	46	–	–	39	79%
	83	TPF		37	–	–	42	80%

*Abbreviations*: *CT* chemotherapy, *RT* radiotherapy, *SC* squamous cell carcinoma, *AC* adenocarcinoma, *pCR* pathologic complete response, *OS* overall survival, *PF* cisplatin plus 5-fluorouracil, *TP* paclitaxel plus cisplatin, *TPF* paclitaxel plus cisplatin plus 5-fluorouracil

In 2008, the Radiation Therapy Oncology Group (RTOG) reported a phase 2 randomized control study called RTOG 0113, which compared two PTX-based regimens used in induction chemotherapy followed by CCR to determine which regimen could achieve a better 1-yr OS and surpassed the historical result of RTOG 9405 [14, 15]. In this study, patients were randomly assigned to two arms. The patients in Arm A received induction chemotherapy with 5-FU 700 mg/m^2^, d1–5, DDP 15 mg/m^2^, d1–5, and PTX 200 mg/m^2^, civ 24 h, q4w * 2 and then CCR with 5-FU 300 mg/m^2^, civ 96 h, and PTX 50 mg/m^2^, d1, qw * 5. The patients in Arm B received induction chemotherapy with PTX 175 mg/m^2^, d1, and DDP 75 mg/m^2^, d1, q3w * 2 and then CCR with DDP 30 mg/m^2^, and PTX 60 mg/m^2^, civ 96 h, qw * 5. Both arms received 50.4 Gy of radiation. Seventy-two patients were assessable in the study, 37 patients in arm A and 35 patients in arm B. In arm A, the median survival time was 28.7 months, the 1-yr and 2-yr OS rates were 76% and 56%, respectively, the incidence rates of grade 3 and 4 toxicities were 54% and 27%, respectively, and treatment-related death was 3%. In arm B, the median survival time was 14.9 months, the 1-yr and 2-yr OS rates were 69% and 37%, respectively, the incidence rates of grade 3 and 4 toxicities were 43% and 40%, respectively, and treatment-related death was 6%. Although the 1-yr OS in Arm A was higher than that in RTOG 9405 (76% vs. 66%), it did not meet the 1-yr survival rate goal.

## Rationale for the trial

Based on this evidence, we designed this phase 3 study to assess whether the PTX-based regimen was better than the PF regimen in CCR. The CCR regimen used in the control group referred to the PF regimen used in RTOG 8501 with some modifications, while the CCR regimen used in the experimental group referred to the TF regimen designed by MDACC in RTOG 0113.

## Methods/design

### Design

**Fig. 1:**
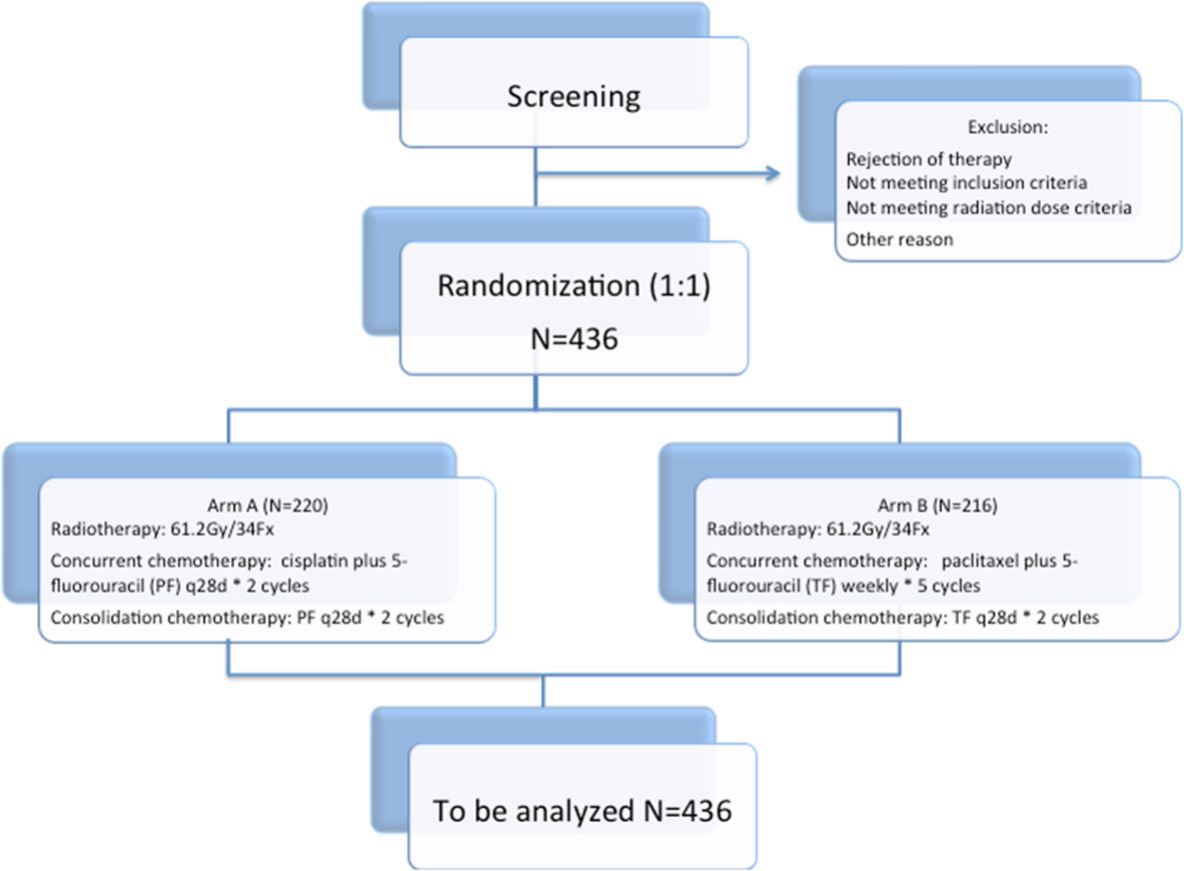
Trial diagram of the ESO-Shanghai 1 trial. 436 esophageal squamous cell carcinoma patients are randomized to either Arm A (concurrent chemoradiotherapy with the PF regimen for 2 cycles followed by consolidation chemotherapy for 2 cycles) or to Arm B (concurrent chemoradiotherapy with a weekly TF regimen for 5 weeks followed by consolidation chemotherapy for 2 cycles)

The ESO-Shanghai 1 trial is a prospective multicenter randomized phase 3 study in which patients are randomized to either CCR with the PF regimen for 2 cycles followed by consolidation chemotherapy for 2 cycles or to CCR with a weekly TF regimen for 5 weeks followed by consolidation chemotherapy for 2 cycles (Fig. 1).

### Objectives

Primary endpoints

- 3-yr OS
  ◦ Defined in the intent-to-treat population.
  ◦ Time from the start of study treatment (Day 1) to death from any cause.

Secondary endpoints

- Disease progression-free survival
  ◦ Defined in the intent-to-treat population.
  ◦ Time from Day 1 to the first event of local failure, metastatic recurrence, progression or death.
  ◦ Progression will be examined by computed tomography (CT), esophageal radiography and/or upper endoscopy
- Local progression-free survival
  ◦ Defined in the intent-to-treat population.
  ◦ Time from Day 1 to the first event of local failure.
  ◦ Local progression will be examined by CT, esophageal radiography and/or upper endoscopy, and is defined as local progression within the irradiated field
- Number and grade of participants with Adverse Events
  ◦ Adverse events will be graded according to the Common Terminology Criteria for Adverse Events version 4.0 [16]

To achieve these objectives, 436 patients will be recruited and randomly allocated in a 1:1 to the two arms by a central randomization center (Fudan University Shanghai Cancer Center, Shanghai, China) stratified by investigator center. SAS will be used to generate a random permutation sequence and produce patient randomization numbers. The data center will register the enrollment, assign a unique identification number to every participant, and reply to the respective investigators. Written informed consent will be obtained from all patients prior to participation in the trial. Patient recruitment will occur at 7 trial centers in China. All participating centers are highly experienced in esophageal radiation oncology, with at least 100 cases performed per year in each center.

### Patient selection

#### Initial diagnosis

1. Endoscopic assessment with biopsy
2. CT scan of chest and ultrasound of abdomen and neck are required (CT/MRIs are acceptable)
3. Fine needle aspirations are required for all superficial lymph node metastases (e.g. supraclavicular lymph node metastases)
4. Full blood count, renal function, and liver function test (bilirubin, AST/ALT, alkaline phosphatase, total protein, albumin, globulin) within 14 days prior to enrolment.
5. Physical examination and history to include height and weight

### Inclusion criteria

Eligible patients must meet all of the following criteria:

1. Joined the study voluntarily and signed informed consent form;
2. Age 18–75 years; both genders
3. Esophageal squamous cell carcinoma confirmed by pathology. No radiotherapy, chemotherapy or other treatments prior to enrollment
4. Local advanced esophageal squamous cell carcinoma (T2N0M0-TxNxM1a, AJCC 2002)
5. Use of an effective contraceptive for adults to prevent pregnancy.
6. No severe abnormal hematopoietic, cardiac, pulmonary, renal, or hepatic function. No immunodeficiency.
7. WBC ≥ 3*10^9^/L, Hemoglobin ≥9 g/dL, Neutrophils ≥1.5 × 10^9^/L, Platelet count (Plt) ≥100*10^9^/L, ALAT and ASAT < 2.5 * ULN, TBIL< 1.5 * ULN, Creatinine < 1.5 *ULN.
8. ECOG 0–2.
9. Life expectancy of more than 3 months.

### Exclusion criteria

Patients meeting any of the following criteria are not eligible for this trial:

1. Complete esophageal obstruction, deep esophageal ulcer, esophageal perforation, or hematemesis.
2. History of radiotherapy or chemotherapy for esophageal cancer.
3. History of surgery within 28 days before Day 1.
4. History of prior malignancies (other than skin basal cell carcinoma or cervical carcinoma in situ with a disease-free survival of at least 3 years).
5. Participation in other interventional clinical trials within 30 days.
6. Pregnant or breast-feeding women or fertile patients who refused to use contraceptives.
7. Drug addiction, alcoholism or AIDS.
8. Uncontrolled seizures or psychiatric disorders.
9. Patients with metastatic disease i.e. M1b according to AJCC 2002.
10. Any other condition which in the investigator’s opinion would not make the patient a good candidate for the clinical trial.

### Study treatment

**Fig. 2:**
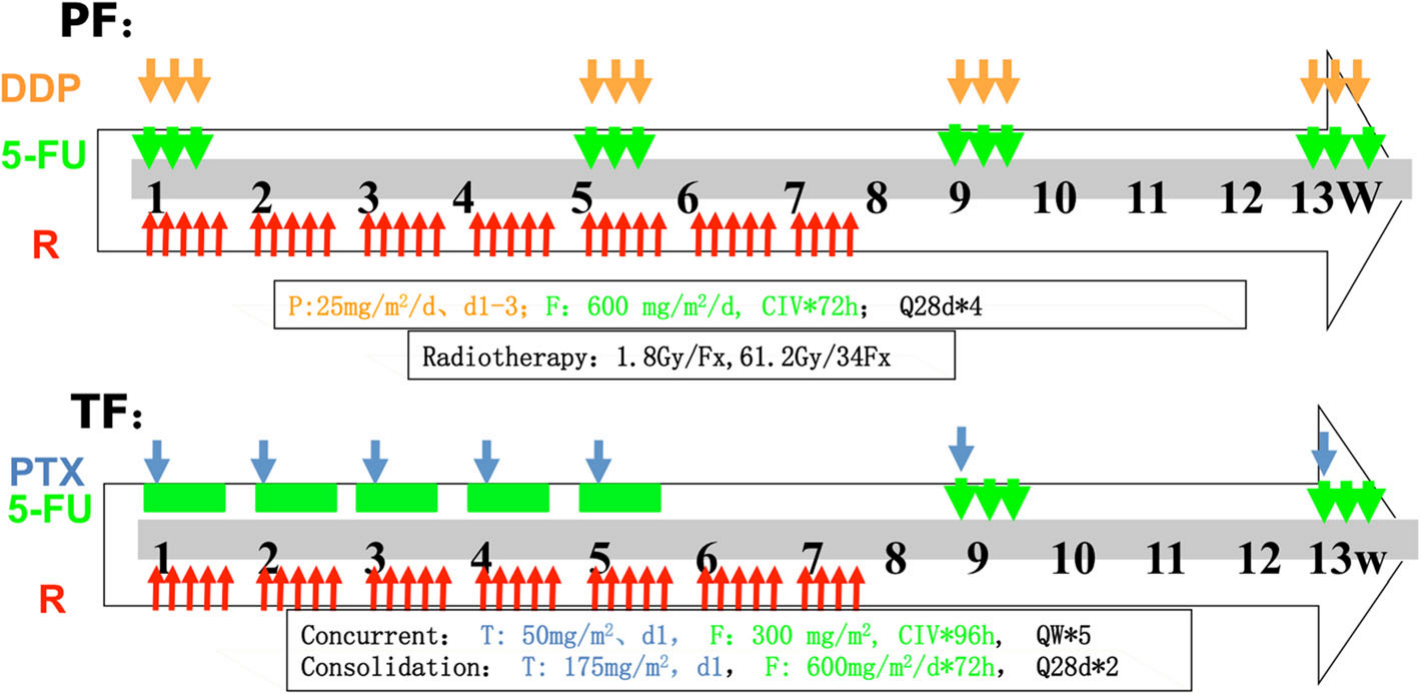
Treatment design of the ESO-Shanghai 1 trial. Patients in both Arms will receive concurrent chemoradiotherapy and consolidation chemotherapy. Radiotherapy will begin on Day 1, concurrent with the beginning of cycle 1 of chemotherapy. (Abbreviations: R, radiotherapy; PTX, paclitaxel; DDP (P), cisplatin; 5-FU (F), 5-fluorouracil; PF, cisplatin plus 5-fluorouracil; TF, paclitaxel plus 5-fluorouracil)

The treatment scheme is shown in Fig. 2. Patients will receive CCR and consolidation chemotherapy. Radiotherapy will begin on Day 1, concurrent with the beginning of cycle 1 of chemotherapy.

### Chemotherapy

#### Cisplatin plus 5-fluorouracil regimen (arm a)

The Arm A consists of 2 cycles of CCR and 2 cycles of consolidated chemotherapy post-radiotherapy. Each cycle of chemotherapy lasts 28 days (4 weeks). The drugs used in Arm A include cisplatin and 5-FU, which are applied intravenously according to the following scheme: 5-FU 1800 mg/m^2^ civ 72 h day 1 and cisplatin 25 mg/m^2^/d day 1–3 every 4 weeks.

#### Paclitaxel plus 5-fluorouracil regimen (arm B)

Arm B consists of 5 cycles of weekly CCR and 2 subsequent cycles of monthly consolidation chemotherapy post-radiotherapy. Each cycle lasts 7 days (1 week) in concurrent chemotherapy and 28 days (4 weeks) in consolidation chemotherapy. The drugs used in Arm B include PTX and 5-FU, which are applied intravenously according to the following scheme: 5-FU 300 mg/m^2^ civ 96 h day 1 and PTX 50 mg/m^2^/d day 1 every week in concurrent chemotherapy and 5-FU 1800 mg/m^2^ civ 72 h day 1 and PTX 175 mg/m^2^/d day every 4 weeks.

#### Chemotherapy interruption and dose modifications

Chemotherapy should be delayed in the conditions as follows:

- ANC < 1.5 × 10^9^/L
- Plt < 80 × 10^9^/L
- ≥Grade 3 nonhematological toxicities (nausea, vomiting and alopecia excluded)

Up to a 2-week delay of chemotherapy is allowed, or the chemotherapy will be terminated. The cycle should be administered only if the patient has recovered from toxicity (ANC ≥1.5 × 10^9^/L, Plt ≥80 × 10^9^/L and <Grade 3 non-hematological toxicities).

The chemotherapy dose should be modified according to the worst toxicity observed during the previous cycle. Chemotherapy should be administered with 25% dose reduction if ANC < 0.5 × 10^9^/L, Plt < 25 × 10^9^/L or ≥Grade 3 non-hematological toxicities. In the case of both ANC < 0.5 × 10^9^/L and Plt < 25 × 10^9^/L, the chemotherapy should be terminated. Modifications up to 2 times are allowed, or chemotherapy will be terminated.

### Radiotherapy (both arms)

Radiotherapy will be delivered in both arms with photons (6 MV) to a total dose of 61.2 Gy in 34 fractions. Patients will be treated 5 days per week at 1.8 Gy/d. Intensity modulated radiotherapy based on CT simulation planning system with 5-mm-thick scan slice throughout the entire neck and thorax is required. We will use involved-field irradiation in this study.

#### Gross tumor volume (GTV)

The GTV is defined as visible esophageal tumor and metastasis lymph nodes based on the imaging of endoscopic ultrasound, esophageal radiography or CT scan (whichever is larger). The criteria for metastatic lymph nodes are as follows: pathologic confirmation or short axis of ≥10 mm in the mediastinum or cervix, short axis of ≥5 mm in the tracheoesophageal groove, or histologically proven metastatic by puncture.

#### Clinical target volume (CTV)

The CTV is defined by the GTV grown manually by 30 mm in the superior-inferior direction for primary tumor and 0 mm for metastatic lymph nodes and 0 mm circumferentially.

#### Planning target volume (PTV)

The PTV is defined as a further 1-cm expansion added to the CTV in all directions. The field next to the spinal cord could be slightly changed to reduce the exposure of the spinal cord.

Tissue inhomogeneity correction is adopted in the planning system. The criteria of dose distribution are as follows: 95% of the PTV to receive ≥99% of the prescribed dose; 99% of the PTV to receive ≥95% of the prescribed dose; <2 cm^3^ of the PTV to receive ≥120% of the prescribed dose; <1 cm^3^ of the PTV to receive ≥110% of the prescribed dose. The highest and lowest dose points inside the PTV should be recorded.

#### Normal organ contouring and dose restrictions

Normal organs, including the spinal cord, heart, right lung, and left lung, should be contoured on each slice of the planning CT with no planning margin. The spinal cord dose constraint cannot be exceeded for any reason. The heart contours should extend from the beginning of the right atrium and right ventricle (pulmonary artery trunk, ascending main aorta and superior vena cava were excluded) down to the apex of the heart. The lung volume is defined as the total lung minus PTV.

The priority order of consideration of normal organ dose restrictions is as follows:

1. Spinal cord: The highest dose point must be less than 45 Gy.
2. Lung: The volume of the lung (PTV excluded) receiving 20 Gy must be equal to or less than 30% of the total lung volume, and the mean lung dose must be equal to or less than 15 Gy at the same time.
3. Heart: The mean dose must be less than 40Gy.

#### Dose modifications

It is strongly recommended that the normal organ dose constraints should not be exceeded. If any dose constraint needs to be exceeded to achieve adequate coverage of the PTV, the physician will decide whether the dose should be modified, or the patient should be excluded from the trial. The acceptable violations of dose modifications are as follows: 92–95% of the PTV to receive ≥99% of the prescribed dose; the normal organ dose restrictions (except the spinal cord) exceeding 5%–10%.

#### Radiotherapy delay

Radiotherapy should be delayed until recovery from the toxicities to no more than grade 2 in the conditions as follows:

- WBC < 2.0 × 10^9^/L or ANC < 1.0 × 10^9^/L
- Plt < 50 × 10^9^/L
- Grade 3 or higher non-hematological toxicity

Radiotherapy should be delayed until complete recovery if infection occurs with fever over 38.5 °C. Up to a 2-week delay of radiotherapy is allowed, or the radiotherapy will be terminated.

#### Radiotherapy quality assurance

The first 3 patients’ IMRT planning CT of each participating institution will be sent to Fudan University Shanghai Cancer Center for centrally quality assurance to demonstrate that the center complies with the specific study requirements for delineation, planning and dose distribution.

### Statistical analysis

With a global alpha risk of 5% and 80% power, an accrual period of 48 months and a minimum follow-up of 36 months, and 6% patient loss, the inclusion of 436 patients (218 in each arm) will be necessary to demonstrate an improvement of 12% in overall survival at 3 years (from 30% in the PF arm to 42% in the TF arm, based upon the results of RTOG 8501 clinical trial and a phase 2 study) [1, 17]. The studied will be terminated when 293 events occur. The median overall survival will be using the Kaplan-Meier method, and the log-rank test will be used to compare the overall survival among treatment arms.

## Discussion

Although the RTOG 8501 showed a better OS in the CCR group with the PF regimen, the results revealed that the PF regimen was not an optimal regimen in CCR with only 30% patients surviving over 3 years and 44% and 20% patients developing severe and life-threatening side effects, respectively [1, 18]. In 2001, MDACC designed a new regimen with continuous infusion of 5-fluorouracil and weekly PTX combined with radiotherapy in the treatment of EC patients [19]. The results showed that this new regimen was well tolerated, with only one patient with grade 2 nausea and vomiting who had a delay of therapy. According to these lines of evidence, we initiated this prospective multicenter randomized phase 3 trial. The aim of this phase 3 trial is to determine whether the TF regimen could replace the standard PF regimen for inoperable ESCC patients. An OS benefit of 12% at 3 years should be expected in the TF group to achieve this goal. There are some limitations in this phase 3 trial. First, we did not stratify the patients according to the status of lymph node metastasis. Since this trial is randomized and the sample size is large enough, the influence of this limitation may be reduced. Second, we used the data of the results of RTOG 8501, which was conducted nearly 30 years ago, to estimate the 3-yr OS in the PF group and calculate the sample size. Because of the improvement in radiotherapy technology, the 3-yr OS of the control group may be underestimated.

## Abbreviations

5-FU: 5-fluorouracil
AC: Adenocarcinoma
AUC: Area under the curve
CBP: Carboplatin
CCR: Concurrent chemoradiotherapy
CR: Complete response
CT: Computed tomography
CTV: Clinical target volume
DDP: Cisplatin
EC: Esophageal carcinoma
ESCC: Esophageal squamous cell carcinoma
GTV: Gross tumor volume
MDACC: University of Taxas M.D. Anderson Cancer Center
OS: Overall survival
pCR: Pathologic complete response
PF: Cisplatin combined with 5-fluorouracil continuous infusion
PTV: Planning target volume
PTX: Paclitaxel
RTOG: Radiation therapy oncology group
SC: Squamous cell carcinoma
TF: Paclitaxel plus 5-fluorouracil
TP: Paclitaxel plus cisplatin
TPF: Paclitaxel plus cisplatin plus 5-fluorouracil

## References

[1] Cooper JS, Guo MD, Herskovic A, Macdonald JS, Martenson JA, Al-Sarraf M, Byhardt R, Russell AH, Beitler JJ, Spencer S (1999). Chemoradiotherapy of locally advanced esophageal cancer: long-term follow-up of a prospective randomized trial (RTOG 85-01). Radiation therapy oncology group. JAMA.

[2] Ajani JA, Ilson DH, Daugherty K, Pazdur R, Lynch PM, Kelsen DP (1994). Activity of taxol in patients with squamous cell carcinoma and adenocarcinoma of the esophagus. J Natl Cancer Inst.

[3] Ilson DH, Wadleigh RG, Leichman LP, Kelsen DP (2007). Paclitaxel given by a weekly 1-h infusion in advanced esophageal cancer. Ann Oncol.

[4] Milas L, Milas MM, Mason KA (1999). Combination of taxanes with radiation: preclinical studies. Semin Radiat Oncol.

[5] Tishler RB, Schiff PB, Geard CR, Hall EJ (1992). Taxol: a novel radiation sensitizer. Int J Radiat Oncol Biol Phys.

[6] van Rijn J, van den Berg J, Meijer OW (1995). Proliferation and clonal survival of human lung cancer cells treated with fractionated irradiation in combination with paclitaxel. Int J Radiat Oncol Biol Phys.

[7] Milas L, Hunter N, Mason KA, Milross C, Peters LJ (1995). Tumor reoxygenation as a mechanism of taxol-induced enhancement of tumor radioresponse. Acta Oncol.

[8] Milas L, Hunter NR, Mason KA, Milross CG, Saito Y, Peters LJ (1995). Role of reoxygenation in induction of enhancement of tumor radioresponse by paclitaxel. Cancer Res.

[9] Kelsey CR, Chino JP, Willett CG, Clough RW, Hurwitz HI, Morse MA, Bendell JC, D'Amico TA, Czito BG (2007). Paclitaxel-based chemoradiotherapy in the treatment of patients with operable esophageal cancer. Int J Radiat Oncol Biol Phys.

[10] Weiner LM, Colarusso P, Goldberg M, Dresler C, Coia LR (1997). Combined-modality therapy for esophageal cancer: phase I trial of escalating doses of paclitaxel in combination with cisplatin, 5-fluorouracil, and high-dose radiation before esophagectomy. Semin Oncol.

[11] Wright CD, Wain JC, Lynch TJ, Choi NC, Grossbard ML, Carey RW, Moncure AC, Grillo HC, Mathisen DJ (1997). Induction therapy for esophageal cancer with paclitaxel and hyperfractionated radiotherapy: a phase I and II study. J Thorac Cardiovasc Surg.

[12] Adelstein DJ, Rice TW, Rybicki LA, Larto MA, Ciezki J, Saxton J, DeCamp M, Vargo JJ, Dumot JA, Zuccaro G (2000). Does paclitaxel improve the chemoradiotherapy of locoregionally advanced esophageal cancer? A nonrandomized comparison with fluorouracil-based therapy. J Clin Oncol.

[13] Thirion P, Piedbois Y (2001). Preoperative chemoradiotherapy using taxanes for locally advanced esophageal carcinoma. J Clin Oncol.

[14] Ajani JA, Winter K, Komaki R, Kelsen DP, Minsky BD, Liao Z, Bradley J, Fromm M, Hornback D, Willett CG (2008). Phase II randomized trial of two nonoperative regimens of induction chemotherapy followed by chemoradiation in patients with localized carcinoma of the esophagus: RTOG 0113. J Clin Oncol.

[15] Minsky BD, Pajak TF, Ginsberg RJ, Pisansky TM, Martenson J, Komaki R, Okawara G, Rosenthal SA, Kelsen DP (2002). INT 0123 (radiation therapy oncology group 94-05) phase III trial of combined-modality therapy for esophageal cancer: high-dose versus standard-dose radiation therapy. J Clin Oncol.

[16] Van Meter EM, Garrett-Mayer E, Bandyopadhyay D (2012). Dose-finding clinical trial design for ordinal toxicity grades using the continuation ratio model: an extension of the continual reassessment method. Clinical trials.

[17] CHEN Y, Ai T, Xia Y, Liu Q, Zhang J, Zhao K (2016). Results of a phase II study of concurrent 5-fluorouracil/paclitaxel plus radiotherapy in patients with carcinoma of the esophagus. China Oncol.

[18] Herskovic A, Martz K, al-Sarraf M, Leichman L, Brindle J, Vaitkevicius V, Cooper J, Byhardt R, Davis L, Emami B (1992). Combined chemotherapy and radiotherapy compared with radiotherapy alone in patients with cancer of the esophagus. N Engl J Med.

[19] Schnirer II, Komaki R, Yao JC, Swisher S, Putnam J, Pisters PW, Roth JA, Ajani JA (2001). Pilot study of concurrent 5-fluorouracil/paclitaxel plus radiotherapy in patients with carcinoma of the esophagus and gastroesophageal junction. Am J Clin Oncol.

[20] van der Gaast A, Kok TC, Vos R, Kerkhofs L, Splinter TA (1997). A phase I dose finding study of a biweekly schedule of a fixed dose of cisplatin with increasing doses of paclitaxel in patients with advanced esophageal cancer. Semin Oncol.

[21] Kelsen D, Ginsberg R, Bains M, Cooper J, Arquette M, Forastiere AA, Ilson D (1997). A phase II trial of paclitaxel and cisplatin in patients with locally advanced metastatic esophageal cancer: a preliminary report. Semin Oncol.

[22] Polee MB, Eskens FA, van der Burg ME, Splinter TA, Siersema PD, Tilanus HW, Verweij J, Stoter G, van der Gaast A (2002). Phase II study of bi-weekly administration of paclitaxel and cisplatin in patients with advanced oesophageal cancer. Br J Cancer.

[23] Huang J, Cai RG, Meng PJ, Zhang MJ, Cui CX, Yang L, Chu DT, Sun Y, Wang JW (2004). Phase II study of paclitaxel and cisplatin for advanced squamous-cell carcinoma of esophagus. Zhonghua Zhong Liu Za Zhi.

[24] Ilson DH, Ajani J, Bhalla K, Forastiere A, Huang Y, Patel P, Martin L, Donegan J, Pazdur R, Reed C (1998). Phase II trial of paclitaxel, fluorouracil, and cisplatin in patients with advanced carcinoma of the esophagus. J Clin Oncol.

[25] Lin CC, Yeh KH, Yang CH, Hsu C, Tsai YC, Hsu WL, Cheng AL, Hsu CH (2007). Multifractionated paclitaxel and cisplatin combined with 5-fluorouracil and leucovorin in patients with metastatic or recurrent esophageal squamous cell carcinoma. Anti-Cancer Drugs.

[26] Safran H, Gaissert H, Akerman P, Hesketh PJ, Chen MH, Moore T, Koness J, Graziano S, Wanebo HJ (2001). Paclitaxel, cisplatin, and concurrent radiation for esophageal cancer. Cancer Investig.

[27] Bains MS, Stojadinovic A, Minsky B, Rusch V, Turnbull A, Korst R, Ginsberg R, Kelsen DP, Ilson DH (2002). A phase II trial of preoperative combined-modality therapy for localized esophageal carcinoma: initial results. J Thorac Cardiovasc Surg.

[28] Urba SG, Orringer MB, Ianettonni M, Hayman JA, Satoru H (2003). Concurrent cisplatin, paclitaxel, and radiotherapy as preoperative treatment for patients with locoregional esophageal carcinoma. Cancer.

[29] van Meerten E, Muller K, Tilanus HW, Siersema PD, Eijkenboom WM, van Dekken H, Tran TC, van der Gaast A (2006). Neoadjuvant concurrent chemoradiation with weekly paclitaxel and carboplatin for patients with oesophageal cancer: a phase II study. Br J Cancer.

[30] Meluch AA, Greco FA, Gray JR, Thomas M, Sutton VM, Davis JL, Kalman LA, Shaffer DW, Yost K, Rinaldi DA (2003). Preoperative therapy with concurrent paclitaxel/carboplatin/infusional 5-FU and radiation therapy in locoregional esophageal cancer: final results of a Minnie pearl cancer research network phase II trial. Cancer J.

[31] Lin CC, Hsu CH, Cheng JC, Wang HP, Lee JM, Yeh KH, Yang CH, Lin JT, Cheng AL, Lee YC (2007). Concurrent chemoradiotherapy with twice weekly paclitaxel and cisplatin followed by esophagectomy for locally advanced esophageal cancer. Ann Oncol.

[32] Jatoi A, Martenson JA, Foster NR, McLeod HL, Lair BS, Nichols F, Tschetter LK, Moore DF, Fitch TR, Alberts SR (2007). Paclitaxel, carboplatin, 5-fluorouracil, and radiation for locally advanced esophageal cancer: phase II results of preliminary pharmacologic and molecular efforts to mitigate toxicity and predict outcomes: north central cancer treatment group (N0044). Am J Clin Oncol.

[33] Roof KS, Coen J, Lynch TJ, Wright C, Fidias P, Willett CG, Choi NC (2006). Concurrent cisplatin, 5-FU, paclitaxel, and radiation therapy in patients with locally advanced esophageal cancer. Int J Radiat Oncol Biol Phys.

